# Inflammation and Oxidative Stress in Obesity-Related Glomerulopathy

**DOI:** 10.1155/2012/608397

**Published:** 2012-04-05

**Authors:** Jinhua Tang, Haidong Yan, Shougang Zhuang

**Affiliations:** ^1^Department of Nephrology, Shanghai East Hospital, Tongji University School of Medicine, Shanghai 200120, China; ^2^Department of Medicine, Alpert Medical School of Brown University, Rhode Island Hospital-Middle House 301, 593 Eddy Street, Providence, RI 02903, USA

## Abstract

Obesity-related glomerulopathy is an increasing cause of end-stage renal disease. Obesity has been considered a state of chronic low-grade systemic inflammation and chronic oxidative stress. Augmented inflammation in adipose and kidney tissues promotes the progression of kidney damage in obesity. Adipose tissue, which is accumulated in obesity, is a key endocrine organ that produces multiple biologically active molecules, including leptin, adiponectin, resistin, that affect inflammation, and subsequent deregulation of cell function in renal glomeruli that leads to pathological changes. Oxidative stress is also associated with obesity-related renal diseases and may trigger the initiation or progression of renal damage in obesity. In this paper, we focus on inflammation and oxidative stress in the progression of obesity-related glomerulopathy and possible interventions to prevent kidney injury in obesity.

## 1. Introduction

Obesity has become a heavy public health problem in the United States, with a prevalence among adults increasing to 32% from 13% between the 1960s and 2004 [1]. Currently, 66% of adults and 16% of children and adolescents are overweight or obese [1]. Although obesity has long been recognized as an independent risk factor for cardiovascular diseases and diabetes mellitus, newer research points to obesity as an important risk factor for chronic kidney diseases (CKDs) [2–4]. In 1974, Weisinger et al. [5] firstly reported that massive obese patients developed nephrotic-range proteinuria. Subsequent studies confirmed that obesity could induce renal injury, namely, obesity-related glomerulopathy (ORG) [6–8]. A large-scale clinicopathologic study including 6818 renal biopsies from 1986 to 2000 revealed a progressive increase in biopsy incidence of ORG from 0.2% in 1986–1990 to 2.0% in 1996–2000 [8]. The tenfold increase in incidence of ORG over 15 years suggests a newly emerging epidemic [8].

The clinical characteristics of subjects with ORG typically manifest with nephrotic or subnephrotic proteinuria, accompanied by renal insufficiency [8–10]. Histologically, ORG presents as focal segmental glomerulosclerosis (FSGS) and glomerular hypertrophy or glomerular hypertrophy alone and relatively decreased podocyte density and number and mild foot process fusion [8, 11, 12]. Clinically, it is distinguished from idiopathic FSGS (I-FSGS) by its lower incidence of nephrotic syndrome, more benign course, and slower progression of proteinuria and renal failure [8, 11].

ORG is an increasing cause of end-stage renal disease (ESRD). The pathophysiology of ORG remains incompletely understood. Potential mechanisms by which obesity affects renal physiology include altered renal hemodynamics, insulin resistance, hyperlipidemia, activation of renin-angiotensin-aldosterone system (RAAS), inflammation, and oxidative stress. Increases in both glomerular filtration rate (GFR) and renal plasma flow (RPF) were observed in obese subjects and animals [13, 14]. This likely occurs because of afferent arteriolar dilation as a result of proximal salt reabsorption, coupled with efferent renal arteriolar vasoconstriction as a result of elevated angiotensin II (AngII) [15]. These effects may contribute to hyperfiltration, glomerulomegaly, and later focal glomerulosclerosis [8, 9]. Insulin resistance can raise the transcapillary pressure gradient and cause hydrostatic pressure and hyperfiltration by reducing norepinephrine-induced efferent arteriolar constriction [16], leading to glomerular hypertrophy and sclerosis. Hyperinsulinemia also has been shown to stimulate the synthesis of growth factors such as insulin-like growth factor- (IGF-) 1 and IGF-2 and transforming growth factor-*β*_1 _(TGF-*β*_1_), which accelerate production of extracellular matrix and promote glomerular hypertrophy and sclerosis [17, 18]. Hyperlipidemia may promote glomerulosclerosis through mechanisms that involve engagement of low-density lipoprotein receptors on mesangial cells, direct podocyte toxicity, oxidative cellular injury, macrophage chemotaxis, and increase renal expression of sterol regulatory element-binding proteins (SREBP-1 and SREBP-2), resulting in the renal accumulation of cholesterol and triglycerides and together with significant renal increase of fibrogenic cytokines [19, 20]. Adipose tissue is the major source of the components of RAAS. Obese subjects usually have increases in plasma renin activity, angiotensinogen, angiotensin-converting enzyme activity, and circulating AngII, which trigger or promote renal damage by renal hemodynamic changes and nonhemodynamic pathways such as hyperinsulinemia, oxidative stress, and inflammation [21–23]. Inflammatory abnormalities and oxidative stress are characteristic findings of obesity and play important roles in the renal damage associated with obesity, which will be discussed in detail in the following.

## 2. Inflammation in Obesity-Related Glomerulopathy

Recent studies have demonstrated that obesity causes chronic low-grade systemic inflammation and thus contributes to the development of systemic metabolic dysfunction that is associated with obesity-related disorders and renal disease [24–27]. Levels of some inflammatory markers and cytokines such as C-reactive protein (CRP), tumor necrosis factor-*α* (TNF-*α*), interleukin-6 (IL-6), and macrophage migration inhibitory factor (MIF) are elevated, whereas concentrations of adiponectin, a protein hormone that exerts anti-inflammatory activities, are reduced in obesity [28–34].

### 2.1. Leptin

Leptin is a 16-kDa-peptide hormone encoded by obese (ob) gene that is mainly produced by adipose tissue. Leptin serves as a regulator of energy balance by binding to the full-length leptin receptors—obese receptor b (Ob-Rb) in the hypothalamus, leading to reduction in food intake and elevation in temperature and energy expenditure [35]. Leptin receptors can be classified as secreted-forms (Ob-Re), short-forms (ob-Ra, c, d, and f) mainly expressed in peripheral tissue, and long-forms (ob-Rb) predominantly expressed in hypothalamus. The kidney expresses abundant concentrations of the truncated isoform of the leptin receptor Ob-Ra, but only a small amount of the full-length receptor Ob-Rb [36]. Leptin production is associated with increased size of adipocytes and is positively correlated with the body mass index (BMI) [37]. Increased circulating leptin, a marker of leptin resistance, is common in obesity. Obesity-induced leptin resistance injures numerous peripheral tissues including kidney, liver, myocardium, and vasculature [36, 38]. Leptin results in the development of renal disease by binding to its specific receptors in renal endothelial cells and mesangial cells. In glomerular endothelial cells, leptin stimulates cellular proliferation, TGF-*β*_1_ synthesis, and type IV collagen production [36, 39]. In mesangial cells, leptin upregulates synthesis of the TGF-*β* type II receptor, but not TGF-*β*_1_, and stimulates glucose transport and type I collagen production through signal transduction pathways involving phosphatidylinositol-3-kinase [40]. Leptin also stimulates hypertrophy, but not proliferation in cultured rat mesangial cells [41]. However, both those cell types increase their expression of extracellular matrix in response to leptin. Transgenic mice with leptin overexpression demonstrated an increase in collagen type IV and fibronectin mRNA in the kidney [41]. Leptin is involved in the development of glomerulosclerosis through a paracrine TGF-*β* pathway (between glomerular endothelial and mesangial cells) that promotes the deposition of extracellular matrix, proteinuria, and, eventually, glomerulosclerosis [39]. Infusion of leptin into normal rats for 3 weeks fosters the development of focal glomerulosclerosis and proteinuria [36].

 Leptin also has proinflammatory actions through its interaction with mediators of innate and adaptive immunity and CRP [38]. Leptin regulates components of innate and adaptive immunity, including T lymphocytes and monocytes/macrophages [42, 43]. Central leptin administration in ob/ob mice accelerates renal macrophage infiltration through the melanocortin system [44]. Leptin stimulates central T-cell production and a peripheral shift in favor of T helper (Th) 1 adaptive immune responses (proinflammatory) as opposed to Th2 responses (anti-inflammatory) [38]. Leptin has been shown to modulate adaptive immunity by enhancing T-cell survival and stimulating production of proinflammatory cytokines such as IFN-*γ* and IL-2 [45]. Leptin also has structural and functional resemblance to proinflammatory cytokines, such as IL-6 [42], and may modulate CRP, a leptin-interacting protein [46].

Therefore, these direct and indirect effects of leptin on the kidney, including stimulating cellular proliferation and hypertrophy, increasing extracellular matrix expression, and exhibiting proinflammatory activities, may partially explain obesity-related kidney disease.

### 2.2. Adiponectin

Adiponectin is a 30 kDa adipocyte-derived protein hormone encoded by the adipose most abundant gene transcript 1 (APM1), [47] which plays a role in the suppression of inflammation-associated metabolic disorders. Two distinct adiponectin receptors, AdipoR1 and R2, have been cloned [48]. AdipoR1 is expressed ubiquitously while AdipoR2 is most abundant in the liver. Adiponectin is highly abundant in human serum, but its level is decreased in most obese animal and human subjects, particularly in those with visceral obesity [49–51]. Recent clinical studies show a negative association of adiponectin in obese patients, [52, 53] suggesting that adiponectin may play a key role in the development of obesity-related albuminuria and alteration of renal function.

Studies with the adiponectin knockout mouse provide evidence that adiponectin can regulate podocyte function and thus contribute to the initial development of albuminuria [37, 53]. Sharma et al. showed that knockout of adiponectin in mice increased albuminuria and caused fusion of podocyte foot processes [53]. In cell culture studies with podocytes, incubation with adiponectin potently decreased permeability to albumin, induced translocation of zona occludens-1 (ZO-1) to the plasma membrane, and reduced the renal predominant NADPH oxidase Nox 4, largely via a 5′-AMP-activated-protein kinase- (AMPK-) dependent pathway. Treatment of the adiponectin knockout mice with exogenous adiponectin was able to decrease albuminuria and improve podocyte morphology [53]. Chronic hyperadiponectinemia significantly alleviated the progression of proteinuria in early-stage diabetic nephropathy by several mechanisms. It led to an increase in nephrin expression, improvement of the endothelial dysfunction due to decreases in endothelin 1 (ET-1) and plasminogen activator inhibitor 1 (PAI-1), and an increase in endothelial nitric oxide synthase (eNOS) expression in the renal cortex [54].

Recent studies suggest that adiponectin exerts anti-inflammatory effects by suppressing TNF-*α*-induced activation of nuclear factor-*κ*B (NF-*κ*B) in human aortic endothelial cells and aortic smooth muscle cells through inhibition of I*κ*B phosphorylation [55, 56] and inhibition of vascular cell adhesion molecule 1 (VCAM-1) and intercellular adhesion molecule 1 (ICAM-1) expression [57], thereby reducing monocyte adhesion and macrophage-induced cytokine production [58] and CRP expression in human adipose tissue [59]. Human adipose tissue expressed CRP, which was negatively correlated with adiponectin expression in adipose tissue [59]. Low levels of adiponectin are associated with higher levels of highly-sensitive C-reactive protein (hs-CRP) and IL-6 [49], two inflammatory mediators that are involved in the initiation and progression of atherosclerosis and renal disease. Therefore, hypoadiponectinemia contributes to development of a low-grade systemic chronic inflammation state, suggesting that hypoadiponectinemia may play a causative role in the systemic and vascular inflammation commonly found in obesity and obesity-related disorders, including renal injury, through its proinflammatory effects.

### 2.3. Resistin

Resistin, also known as adipocyte-specific secretory factor (ADSF) or as found in inflammatory zone (Fizz), is a cysteine-rich 12.5-kDa polypeptide that belongs to a small family called resistin-like molecules (RELMs) [60, 61]. In rodents, resistin is secreted from white adipocytes [62, 63]. In human, it is produced largely by macrophages and expressed in adipose tissue predominantly by nonadipocyte resident inflammatory cells [64–66]. Current evidence suggests that resistin has been variably associated with obesity, insulin resistance, inflammation, and renal dysfunction. Resistin levels are elevated in both genetic and diet-induced animal models of obesity [62, 67]. Studies of obese subjects have frequently noted higher serum levels of resistin as well as direct correlations between resistin level and adiposity as measured by BMI [68, 69]. There has been a link between circulating resistin and low-grade inflammation that accompany obesity [70]. Resistin is associated with elevated CRP and white blood cells, suggesting that the role of resistin may be a component of obesity-related inflammation [70]. It has recently been found that resistin involves in the regulation of proinflammatory cytokine expression. Resistin strongly upregulates IL-6 and TNF-*α* in human peripheral blood mononuclear cells (PBMCs) via NF-*κ*B pathway [71]. Human resistin enhanced secretion of proinflammatory cytokines, TNF-*α* and IL-12 in macrophages by NF-*κ*B-dependent pathway [72]. Studies also show that increased levels of resistin in patients with CKD are associated with declined renal function and inflammation [73, 74]. This suggests that resistin may play an important role in obesity and obesity-associated disease by triggering the release of other proinflammatory cytokines.

### 2.4. Inflammatory Markers

A growing body of evidence indicates that obesity-related glomerulopathy is associated with upregulation of inflammatory mediators [75]. Obesity leads to adipose tissue macrophage infiltration in white adipose tissue and increased levels in proinflammatory cytokines. Several inflammatory mediators released from adipocytes and macrophages, such as TNF-*α*, IL-6, IL-1*β*, CRP, monocyte chemoattractant protein 1 (MCP-1), PAI-1, and MIF, contribute to a low level of chronic inflammatory state in obesity and may be responsible for renal injury in obesity-associated glomerulopathy. An emerging pattern of gene expression was observed in adipose tissue in mice fed high fat-fed diets, indicating a shift toward global upregulation of inflammatory genes, including TNF-*α*, IL-6, and MCP-1 [76].

TNF-*α*, a proinflammatory cytokine, is predominantly produced by macrophages infiltrating adipose tissue [77, 78] and can also be produced by the kidney [79]. This cytokine is involved in the genesis of inflammation and contributes to obesity-associated insulin resistance [80–82]. Within the kidney, AngII, advanced glycation end-products (AGEs), and oxidized low-density lipoprotein (LDL) can stimulate TNF-*α* synthesis from renal cells to initiate local damage [83–86]. A recent study demonstrates that TNF-*α* reduces the expression of Klotho, a protein expressed by renal cells, through an NF-*κ*B-dependent mechanism, which contribute to renal injury [87]. TNF-*α* enhances the expression of PAI-1 in human adipose tissue and plasma PAI-1 levels in obesity subjects and is responsible for reduced fibrinolysis and also a component of extracellular matrix, leading to renal fibrosis and terminal renal failure [88–90]. TNF-*α* also has been shown to induce the expression of MCP-1 via p38 mitogen-activated protein kinase (MAPK) signaling pathway in renal mesangial cells [91]. MCP-1, a key regulator in recruiting monocytes to the glomeruli, may also contribute to renal damage at a later stage of kidney disease in obesity.

IL-6 is another important proinflammatory mediator systemically secreted from adipose tissue and locally produced in the kidney. Studies have demonstrated the positive relationship between BMI and plasma IL-6 concentrations [92–94]. Studies also suggest that IL-6 plays a key role in the development of renal disease. Endogenous IL-6 enhances the degree of renal injury, dysfunction, and inflammation caused by ischemia/reperfusion by promoting the expression of adhesion molecules and subsequent oxidative stress [95]. Transgenic knockout of IL-6 ameliorates renal injury as measured by serum creatinine and histology [96]. Blocking the IL-6 receptor prevents progression of proteinuria and renal lipid deposit as well as the mesangial cell proliferation associated with severe hyperlipoproteinemia [97]. IL-6 also stimulates the synthesis of CRP [98], which is well known as both a marker and important risk factor of atherosclerosis in the general population and CKD patients [99].

MIF was initially described as an immunomodulatory factor isolated from the supernatants of T lymphocytes and was found to inhibit the random migration of macrophages [100]. Subsequent studies have indicated that MIF acts as a proinflammatory cytokine and pituitary-derived hormone that potentiates endotoxemia [101]. MIF also plays a pathogenic role in kidney disease. In a rat model of immunologically induced crescentic antiglomerular basement membrane glomerulonephritis, treatment with anti-MIF antibody reduced proteinuria, prevented the loss of renal function, attenuated histological damage including glomerular crescent formation, inhibited renal leukocytic infiltration and activation, and reduced IL-1*β* expression by both intrinsic kidney cells and macrophages [102]. Thus, MIF is a key mediator of the inflammatory and immune response and plays a pathological role in immune-mediated renal injury.

## 3. Oxidative Stress in Obesity-Related Glomerulopathy

### 3.1. Obesity and Oxidative Stress

Oxidative stress is caused by an imbalance between increased production of reactive oxygen species (ROS) and/or reduced antioxidant activity, leading to oxidative damage to cells or tissue including lipids, proteins and DNA. It is known that oxidative stress is involved in pathological processes of various diseases, such as cancer, diabetes mellitus, hypertension, and cardiovascular disease. Studies have suggested that obesity is associated with increased oxidative stress [103, 104]. Analysis of oxidative markers in obesity subjects indicates that oxidative damage is associated with increased BMI and percentage of body fat [105, 106]. Conversely, parameters of antioxidant capacity are inversely related to the amount of body fat and central obesity [107, 108]. The possible mechanisms of obesity-related oxidative stress include increased oxygen consumption and subsequent production of free radicals derived from the increase in mitochondrial respiration, diminished antioxidant capacity, fatty acid oxidation, lipid oxidizability, and cell injury causing increased rates of free radical formation [104, 109, 110]. It is also reported that the increase in obesity-associated oxidative stress is due to the presence of excessive adipose tissue accumulation [111]. Accumulated adipose tissue generates an immune response leading to the secretion of proinflammatory cytokines, including TNF-*α*, IL-1, and IL-6, which lead to increased generation of ROS. Excessive fat accumulation also stimulates nicotinamide adenine dinucleotide phosphate (NADPH) oxidase activity, which contributes to ROS production [112]. ROS, in return, augmented the expressions of NADPH oxidase (NOX) subunits, including NOX4 and PU.1 in adipocytes, establishing a vicious cycle that augments oxidative stress in white adipose tissue and blood [112].

### 3.2. Oxidative Stress and Inflammation

Oxidative stress in adipocyte seems to be responsible for the low-grade proinflammatory state commonly observed in obesity [113, 114]. Oxidative stress is increasingly viewed as a major upstream component in cell-signaling cascades involved in inflammatory responses, stimulating the expression of proinflammatory cytokines. ROS activate redox-sensitive transcription factors, particularly NF-*κ*B, inducing the release of proinflammatory cytokines and the expression of adhesion molecules and growth factors, including TNF-*α*, IL-6, IL-1*β*, TGF-*β*_1_, connective tissue growth factor, IGF-1, platelet-derived growth factor, and VCAM-1 [115, 116]. H_2_O_2_ stimulates IL-4 and IL-6 gene expression and cytokine secretion by an apurinic/apyrimidinic-endonuclease/redox-factor-1- (APE/Ref-1-) dependent pathway [117]. ROS increased the expression levels of PAI-1, IL-6, and MCP-1 through NADPH oxidase pathway [112]. Oxidized high-density lipoprotein (HDL) enhances proinflammatory properties such as TNF-*α* and MCP-1 in renal mesangial cells partly via CD36 and LDL receptor-1 and via MAPK and NF-*κ*B pathways [118]. Oxidized LDL can stimulate TNF-*α* synthesis from renal cells and initiate local effects of renal damage [85]. Increased ROS production and MCP-1 secretion from accumulated fat may cause infiltration of macrophages and inflammation in adipose tissue of obesity [112]. Moreover, enhanced macrophage migration induces the release of proinflammatory cytokines, which further stimulates the generation of ROS [119, 120]. Therefore, oxidative stress-induced cytokine production is likely to further increase oxidative stress levels, setting a vicious cycle [121] that may promote the progression of kidney damage in obesity.

### 3.3. Oxidative Stress Leads to Renal Injury in Obesity

Oxidative stress has been commonly identified in obesity-related renal diseases and may be the mechanism underlying the initiation or progression of renal injury in obesity [122, 123]. Previous studies suggest that oxidative stress triggers, at an early age, the onset of kidney lesions and functional impairment in Zucker obese (ZO) fa/fa rats, a good model of obesity-related renal disease, in absence of hyperglycaemia, hypertension, and inflammation [124]. ROS play a crucial role in mediating renal injury [125–127]. ROS are highly reactive molecules that oxidize lipids and proteins, cause cellular injury, and promote glomerular and renal tubule injury and associated proteinuria [128].

ROS are produced by various cells, such as vascular cells, inflammatory cells, and renal cells, and have distinct function on different types of cells, such as endothelial dysfunction, inflammatory gene expression, and renal tubule ion transport. A major source for vascular and renal ROS is a Nox family of nonphagocytic NAD(P)H oxidases, including the prototypic Nox2 homolog-based NAD(P)H oxidase, as well as other NAD(P)H oxidases, such as Nox1 and Nox4 [129]. Numerous reports indicate that within the kidney, NAD(P)H oxidase, an enzyme that produces superoxide (O_2_^•−^) by transferring electrons from NADH/NADPH to molecular oxygen and thereby forming O_2_^−^, H^+^, and NAD^+^/NADP^+^, is capable of modulating renal epithelial ion transport [130, 131]. NAP(D)H oxidase-derived ROS can alter renal pressure natriuresis and blood pressure regulation through its effects on renal hemodynamics and renal tubular sodium transport. Recent data suggest that NADPH oxidase-mediated oxidative injury to the proximal tubule, like that seen in the glomerulus, contributes to proteinuria in insulin-resistant states [128].

Oxidative stress also plays an important role in the pathogenesis of renal damage through its effects on vascular biology. ROS are generated by all types of vascular cells, including endothelial, smooth muscle, and adventitial cells. ROS influence vascular cell growth, migration, proliferation, and activation [132, 133]. Physiologically, ROS can mediate cellular function, receptor signals, and immune responses on vascular cells. In pathophysiological condition, ROS contribute to progressive vascular dysfunction and remodeling through oxidative damage caused by decreased nitric oxide (NO) bioavailability, impaired endothelium-dependent vasodilatation and endothelial cell growth, apoptosis or anoikis, endothelial cell migration, and activation of adhesion molecules and inflammatory reactions [134, 135].

## 4. Potential Interventions to Prevent Renal Injury

### 4.1. Anti-Inflammation Therapy

Obesity accelerates the progression of renal injury, associated with augmented inflammation in adipose and kidney tissues [136]. Anti-inflammation therapy might be a potential treatment for renal damage. IL-6 is a key inflammatory molecule in renal diseases. Studies in IL-6 transgenic mice suggested that high concentrations of IL-6 contribute to development of renal injury [137]. Treatment with anti-IL-6 receptor antibody MR16-1 prevented progression of proteinuria, renal lipid deposit, and the mesangial cell proliferation in hypercholesterolemia-induced renal injury [97]. TNF-*α* is another important proinflammatory cytokine in the development of renal diseases. Inhibition of TNF-*α* by etanercept, a TNF-*α* antagonist, also decreased blood pressure and protected the kidney through reduction of renal NF-*κ*B, oxidative stress, and inflammation [138]. TNF-*α* blockade increases renal Cyp2c23 expression and slows the progression of renal damage in salt-sensitive hypertension [139]. TNF-*α* inhibition also reduces renal injury in deoxycorticosterone-acetate- (DOCA-) salt hypertensive rats via suppression of renal cortical NF-*κ*B activity [140]. Adiponectin is an adipose-secreted hormone with anti-inflammatory properties. Treatment of adiponectin-knockout mice with adenovirus-mediated adiponectin results in amelioration of albuminuria, glomerular hypertrophy, and tubulointerstitial fibrosis and reduces the elevated levels of VCAM-1, MCP-1, TNF-*α*, TGF-*β*_1_, collagen type I/III, and NADPH oxidase components [141]. Adiponectin prevents glomerular and tubulointerstitial injury through modulating inflammation and oxidative stress [141].

Excessive fat accumulation contributes to macrophage infiltration in adipose tissue and increased production of proinflammatory cytokines, such as TNF-*α*, IL-8, and IL-6 [142–145]. Consequently, it is possible that weight loss may be a potential method to reduce inflammation. Evidence indicates that weight loss induced by nutritional intervention or gastric surgery markedly improves the systemic and adipose tissue inflammatory states linked to obesity [143, 146, 147]. Studies by gene profiling analysis have shown that caloric restriction-induced weight reduction leads to the regulation of a wide variety of inflammation-related molecules in human adipose tissue [148]. Weight loss globally improves the inflammatory profile of obese subjects through a decrease of proinflammatory factors and an increase of anti-inflammatory molecules in white adipose tissue [148]. Roux-en-Y-Gastric-Bypass- (RYGB-) induced weight loss has been shown to reduce MCP-1, IL-18, IL-6, and TNF-*α* concentrations [149, 150]. A longer-term weight reduction induced by RYGB in corpulence also prevails in regulating circulating cytokine concentrations [151]. Weight loss also ameliorates the low-grade inflammation state that leads to glomerular dysfunction in obesity. In morbidly obese individuals with glomerular hyperfiltration, weight loss by surgical interventions normalizes glomerular filtration rate (GFR) and reduces blood pressure and microalbuminuria [152]. Weight loss improves renal function as shown by reduced levels of serum creatinine and improved creatinine clearance [153].

### 4.2. Antioxidant Intervention

Since ROS play a key role in the pathogenesis of renal injury such as glomerulosclerosis and tubulointerstitial fibrosis, approaches to reduce oxidative stress by antioxidants supplementation, nutritional and surgical interventions may have renoprotective effects. Garcinia protects against obesity-induced nephropathy by attenuating oxidative stress through reduced lipid peroxidation and levels of oxidized LDL [154]. The obese Zucker rat is a good model for studying obesity-related kidney disease because it develops proteinuria, glomerular hypertrophy, and focal segmental glomerulosclerosis [155–157]. Using these rats, it has been demonstrated that nephropathy is associated with oxidative stress, and supplementation with an antioxidant ebselen improved kidney damage by ameliorating proteinuria and renal focal and segmental sclerosis [158]. Chronic ebselen therapy also improved vasculopathy with lipid deposits, tubulointerstitial scarring, and inflammation [158]. Other antioxidants also have renoprotective effects. For example, administration of grape seed proanthocyanidin extract (GSPE), an efficient phytochemical antioxidant, can protect against the nephrotoxicity effects induced by cisplatin and gentamicin [159, 160] and reverse experimental myoglobinuric acute renal failure [161]. Quercetin, a flavonoid that exhibits antioxidant properties in many diseases, could also protect the rat kidney against lead-induced injury and improve renal function [162]. Thus, antioxidants may be a potential therapeutic to prevent the renal damage in ORG.

Nutritional and surgical interventions are additional approaches to reduce oxidative stress and prevent kidney injury in obesity. Caloric restriction and protein restriction reduce free radicals and ROS formation and inhibit accumulation of oxidative biomarkers in animal models [163]. In genetically obese animals, diet restriction can prevent or greatly delay the onset of specific degenerative lesions, in particular glomerulonephritis associated with obesity [164]. Since adipose tissue mass in obesity contributes to oxidative stress, bariatric surgery-induced weight loss also results in decreasing systemic oxidative stress in adiposity [111]. Weight loss induced by diet restriction or bariatric surgery not only improves inflammation state but also reduces oxidative stress state in obesity, which may protect renal function in obesity-related glomerulopathy.

## 5. Conclusion

Obesity causes chronic low-grade inflammation and systemic and local oxidative stress, which may play a pivotal role in the initiation or progression of obesity-associated glomerulopathy. Elevated inflammation in obesity is the result of the production of adipokines and increased inflammatory cytokines and decreased anti-inflammatory factors. Oxidative stress is triggered by an imbalance between increased production of ROS and/or reduced antioxidant activity. Both inflammation and oxidative stress induce damage to renal tubule and glomerulus and result in endothelial dysfunction in the kidney. Therefore, anti-inflammation and antioxidant interventions may be the potential therapies to prevent and treat obesity-related renal diseases.
